# Supplementary material for: ProteinShader: illustrative rendering of macromolecules
Source: BMC Struct Biol. 2009 Mar 30;9:19. doi: 10.1186/1472-6807-9-19 (PMC2672931; doi:10.1186/1472-6807-9-19)
Supplement: Additional file 1 — ProteinShader program without source code. This compressed file contains the complete ProteinShader program including associated libraries, but no source code. A README.txt file gives an overview of the ProteinShader distribution, and the index.html file in the help subdirectory has directions on getting started with the program as well as a set of tutorials. [file 1472-6807-9-19-S1.zip › ProteinShader-beta-0_9_4-binary/help/api/org/proteinshader/gui/components/menubar/package-summary.html]

org.proteinshader.gui.components.menubar (ProteinShader API)


|  |  |  |
| --- | --- | --- |
| **Overview** \| **Package** \| Class \| **Use** \| **Tree** \| **Deprecated** \| **Index** \| **Help** | |  |
| **PREV PACKAGE**   **NEXT PACKAGE** | **FRAMES**    **NO FRAMES**     **All Classes** |  |


---

## Package org.proteinshader.gui.components.menubar

Holds the JMenus used in menu bar above the canvas.

**See:**
  
          **Description**

| **Class Summary** | |
| --- | --- |
| **AboutBox** | A simple dialog box that displays a title, an image (PNG, GIG, or JPEG), and a close button. |
| **BackgroundMenu** | Provides the 'Background' menu of the MainMenuBar, which allows the user to change the background color of the canvas. |
| **FileMenu** | Provides the File menu for the MainMenuBar. |
| **HelpMenu** | Provides an "About" box and help for using the ProteinShader program. |
| **MainMenuBar** | Provides the menu bar to place at the top of the ProteinShader programs's main GUI window. |
| **OrientationMenu** | Provides the Orientation menu for the MainMenuBar. |
| **StyleMenu** | Provides a menu for selecting the style to use when displaying a structure: Cartoon (Tubes or Ribbons) or Atom (Space Filling, Balls-andSticks, or Sticks). |
| **ToolsMenu** | Provides the Tools menu for the MainMenuBar, which can be used to open and close the control panel on the right side of the GUI. |
| **VisibilityMenu** | Provides the Vi menu for the MainMenuBar. |

## Package org.proteinshader.gui.components.menubar Description

Holds the JMenus used in menu bar above the canvas.
